# Integration of Kinase and Calcium Signaling at the Level of Chromatin Underlies Inducible Gene Activation in T Cells

**DOI:** 10.4049/jimmunol.1602033

**Published:** 2017-09-13

**Authors:** Ruth Brignall, Pierre Cauchy, Sarah L. Bevington, Bethany Gorman, Angela O. Pisco, James Bagnall, Christopher Boddington, William Rowe, Hazel England, Kevin Rich, Lorraine Schmidt, Nigel P. Dyer, Mark A. Travis, Sascha Ott, Dean A. Jackson, Peter N. Cockerill, Pawel Paszek

**Affiliations:** * Faculty of Biology, Medicine and Health, University of Manchester, Manchester M13 9PT, United Kingdom;; † Institute of Biomedical Research, College of Medicine and Dentistry, University of Birmingham, Birmingham B15 2TT, United Kingdom;; ‡ Centre for Stem Cells and Regenerative Medicine, King’s College London, London SE1 9RT, United Kingdom;; § Department of Chemistry, Loughborough University, Loughborough LE11 3TU, United Kingdom;; ¶ Manchester Collaborative Centre for Inflammation Research, University of Manchester, Manchester M13 9PT, United Kingdom;; ‖ Wellcome Trust Centre for Cell-Matrix Research, University of Manchester, Manchester M13 9PT, United Kingdom; and; # Warwick Systems Biology Centre, University of Warwick, Coventry CV4 7AL, United Kingdom

## Abstract

TCR signaling pathways cooperate to activate the inducible transcription factors NF-κB, NFAT, and AP-1. In this study, using the calcium ionophore ionomycin and/or PMA on Jurkat T cells, we show that the gene expression program associated with activation of TCR signaling is closely related to specific chromatin landscapes. We find that calcium and kinase signaling cooperate to induce chromatin remodeling at ∼2100 chromatin regions, which demonstrate enriched binding motifs for inducible factors and correlate with target gene expression. We found that these regions typically function as inducible enhancers. Many of these elements contain composite NFAT/AP-1 sites, which typically support cooperative binding, thus further reinforcing the need for cooperation between calcium and kinase signaling in the activation of genes in T cells. In contrast, treatment with PMA or ionomycin alone induces chromatin remodeling at far fewer regions (∼600 and ∼350, respectively), which mostly represent a subset of those induced by costimulation. This suggests that the integration of TCR signaling largely occurs at the level of chromatin, which we propose plays a crucial role in regulating T cell activation.

## Introduction

**FIGURE 1.**
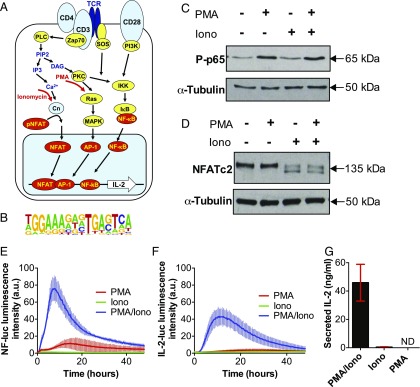
PMA and ionomycin treatment decouple the IKK/MAPK and calcium signaling networks in Jurkat T cells. (**A**) Schematic representation of TCR signaling network. (**B**) Composite consensus binding motif for cooperative binding of NFAT and AP-1 (1). (**C** and **D**) Immunoblotting analyses of NF-κB phospho-p65 (C) and NFATc2 protein expression levels in Jurkat T cells. Cells stimulated with PMA (20 ng/ml), ionomycin (1 μg/ml), or PMA/ionomycin (20 ng/ml PMA/1 μg/ml ionomycin) for 15 and 60 min, respectively. (**E** and **F**) Time series live-cell luminometry analysis of Jurkat T cells expressing reporters of NF-κB–dependent transcriptional activity (E) or IL-2 promoter activity (F) following treatment with PMA, ionomycin, or PMA/ionomycin for 48 h (in triplicates, with error bars indicating SDs). (**G**) Concentration of IL-2, measured by ELISA, secreted by Jurkat T cells treated with PMA/ionomycin (20 ng/ml PMA/1 μg/ml ionomycin), ionomycin (1 μg/ml), or PMA (20 ng/ml). Means (±SDs) of triplicate experiment is shown in red. ND, not detected.

T lymphocytes are vital for mounting efficient immune responses to invading pathogens. When T cells are fully activated, hundreds of immune response genes are induced by up to 1000-fold (1). These responses need to be tightly controlled because any inappropriate responses can lead to proinflammatory immune disorders. Correctly regulated T cell activation is underpinned by signaling events that are initiated by the binding of the cognate Ag to the TCR expressed on the cell surface, and amplified by the CD28 and other costimulatory receptors that act to augment TCR signaling (2–4). A simplified view of the TCR/CD28 signaling network is depicted in Fig. 1A to show the major nodes and targets in this network. However, the signaling machinery that is actually recruited to the immune synapse is far more complex and involves considerable crosstalk between the Ca^2+^ and kinase signaling pathways upstream of the transcription factors (TFs) NFAT, AP-1, and NF-κB (2, 3, 5). TCR activation triggers signaling from ZAP-70 to the enzyme phospholipase Cγ, which cleaves phosphoinisitol phosphate to generate the two critical signaling molecules inositol triphospate and diacly glycerol. This represents a major branchpoint upstream of two critical sets of TFs (Fig. 1A). Inositol triphospate signaling leads to an increase in free intracellular Ca^2+^, which induces calcineurin-mediated dephosphorylation and import of NFAT family TFs into the nucleus (6–8). In parallel, diacly glycerol activates protein kinase C (PKC)–dependent induction of IκB kinase (IKK) and the Ras/Raf/MAPK signaling pathways, leading to the activation of both NF-κB and AP-1, respectively (9, 10). NFAT, NF-κB, and AP-1 cooperate in the activation of gene expression programs that underpin immune responses (8, 11, 12). In particular, this involves the upregulation and secretion of IL-2, which supports the clonal expansion of newly activated T cells, plus many other inducible cytokines such as IL-4, IL-3, and CSF2 (12–23). Studies of these genes revealed that NFAT and AP-1 typically bind cooperatively to composite DNA elements in a specific configuration (11, 21, 24–26).

To better define genomic targets of the TCR signaling network we recently performed a global analysis of inducible DNase I hypersensitive sites (DHSs) in stimulated mouse T cells (1). We defined ∼1000 highly inducible DHSs, representing potential enhancers or promoters, which were highly enriched for binding sites for NFAT and AP-1. One third of these sites contained the composite NFAT/AP-1 motif depicted in Fig. 1B. Additionally, the binding motif for NF-κB was detected in 12% of these inducible DHSs.

The activation of gene expression (27, 28) and the establishment of immunological memory (1, 29–32) in T cells are controlled by TFs that recruit chromatin modifiers and remodelers to transcriptional enhancers and promoters. Specific promoters and enhancers associated with TCR-inducible genes have been shown to undergo rapid changes in DHSs following stimulation, which is indicative of regulatory chromatin rearrangements (18, 33, 34). In particular, NFAT has been extensively associated with alterations in chromatin structure (35–38) and has been shown to be essential in inducing DHS formation within the *IL2* locus (39). However, the activity of NFAT is largely dependent on its ability to bind to DNA cooperatively with AP-1 proteins (15, 21, 24, 25, 40). For example, T cells expressing engineered NFAT proteins that were unable to bind AP-1 displayed diminished effector function and suppression of TCR signaling to inducible genes (40, 41, 42). NFAT/AP-1 complexes bind cooperatively to composite DNA elements present in both proximal and distal enhancer regions of many TCR-inducible genes. Once bound, they typically induce the formation of nucleosome-free DHSs at the regulatory elements that activate gene expression (15, 24, 36, 37). Overall, these studies highlight the critical role of enforced cooperation between NFAT and AP-1 family TFs in maintaining the tight regulation of the T cell gene expression program. In the absence of this cooperativity, TCR activation leads to T cell exhaustion (41).

The recent introduction of transposase-accessible chromatin analysis using sequencing (ATAC-seq) as an alternative to DNase I for mapping nuclease hypersensitive sites provides another high-resolution method of investigating the mechanisms involved in the regulation of gene expression (43). In this study, we employed RNA and ATAC sequencing to define the gene expression patterns and chromatin landscapes associated with TCR signaling. To understand how IKK and MAPK signaling pathways integrate with Ca^2+^ signaling pathways within the TCR regulatory network, we used the calcium ionophore ionomycin and PMA, in combination or alone, to activate Jurkat T cells, a well-established model for studying T cell activation (44). We found that full activation of the core gene expression program associated with T cell activation was dependent on the synergistic stimulation of both signaling networks. These T cell activation programs are established by the rapid remodeling of chromatin upon stimulation, which was found to be associated with the enrichment of composite NFAT/AP-1 elements. Incomplete stimulation, via PMA or ionomycin alone, led to specific gene expression profiles that only partially overlapped with the patterns induced by full activation. Importantly, a strong correlation was found between the stimulus-specific gene expression patterns and the chromatin state.

## Materials and Methods

### Reagents and cell culture

Human Jurkat T cells (A3 clone from American Type Culture Collection) were grown in RPMI 1640 media (Sigma-Aldrich) supplemented with 10% FBS (Life Technologies) and 2 mM l-glutamine (Sigma-Aldrich). Cells were cultured at concentrations between 2 × 10^5^ and 2 × 10^6^ cells/ml and incubated at 37°C and 5% CO_2_. Cells were stimulated with the calcium ionophore ionomycin, derived from *Streptomyces conglobatus* (Sigma-Aldrich), PMA (Sigma-Aldrich), and TNF-α (Calbiochem).

### Development of reporter cell lines

The lentiviral luciferase reporters for IL-2 and NF-κB transcriptional activity were generated by inserting a multiple cloning site into a pRE1x plasmid [a gift from Schibler and colleagues (45)] using *EcoRV* and *Age1* restriction enzymes. The 600-bp proximal promoter of IL-2 was then cloned into the plasmid using the *AscI* restriction enzyme to drive the expression of the luciferase reporter (IL-2–Luc). The reporter for NF-κB (5κB-Luc), which included the *5x* repeat κB consensus sequence was incorporated into the sequence using *Pac1* and *Nhe1* restriction enzymes. Lentivirus production and transduction were carried out as previously described (46).

### Live-cell luminometry

Cells were plated in 24-well white opaque plates (PerkinElmer) in 1 ml of media, prior to incubation with luciferin (0.5 mM; Biosynth, Staad, Switzerland) for at least 4 h before assaying. Live-cell measurements were then collected using a FLUOstar Omega microplate reader (BMG Labtech) with an attached incubator that maintained cells at 37°C and 5% CO_2_. Readings were taken from individual wells every 10 min with an 18-s integration time for up to 48 h. Results are shown as mean fold induction relative to an untreated control ± SD of at least three independent experiments. For statistical analysis, the maximum luminescent intensity for each condition was generated before calculating a *p* value using an unpaired *t* test.

### Detection of IL-2 by ELISA

Jurkat T cells were plated at 5 × 10^5^ in 2 ml of media before being incubated with the appropriate stimuli for 24–48 h. Supernatant was cleared of cells and debris by centrifugation at 300 × *g* for 5 min. The supernatant was then aliquoted and stored at −20°C for future use. Measurement of secreted protein levels was performed using specific human IL-2 ELISA kits (R&D Systems) following the manufacturer’s instructions. Absorbance was measured at 370 nm using a FLUOstar multimode microplate reader. Statistical analysis was performed with a nonparametric one-way ANOVA.

### Western blotting

Cells were lysed with 200 μl of lysis buffer (50 mM Tris-HCl [pH 7.4], 1% Nonidet P-40, 0.5% sodium deoxycholate, 0.1% SDS, 150 mM NaCl, 2 mM EDTA, 50 mM NaF). Samples (18 μl) were then loaded onto gels alongside 8 μl of protein ladder. The proteins were then transferred to nitrocellulose membranes before incubation in blocking buffer (5% [w/v] skimmed milk powder in TBST) for 1 h at room temperature. The blots were then washed in TBST and incubated overnight with anti-NFATc2 (NFAT1) (BD Transduction Laboratories) or anti–phospho-p65 (S536) (Cell Signaling Technology) primary Abs (BD Transduction Laboratories) at 1:5000 and 1:1000 dilution, respectively, in 5% (w/v) BSA in TBST. Blots were washed in TBST and then incubated for a further hour with a 1:2000 dilution of HRP-conjugated anti-IgG (Cell Signaling Technology). After a final TBST wash to remove any unbound Ab, a 1:1 ratio mix of ECL Advance Western blotting detection kit reagents was then applied to the membrane and left for 1 min, before visualizing HRP-conjugated proteins using a Bio-Rad Gel Doc XRS+ system. Membranes were then restained with an anti–α-tubulin primary Ab (Cell Signaling Technology), and the detection process was repeated to produce a loading control.

### RNA isolation and RNA sequencing analysis

Wild-type Jurkat T cells were plated at a density of 5 × 10^6^ cells/ml in 2 ml of media. Total RNA was extracted from cells at appropriate time points after stimulation using the Roche High Pure RNA isolation kit according to the manufacturer’s instructions. The RNA was then quantified using a Nanodrop ND-1000 spectrophotometer (Thermo Fisher Scientific). RNA-seq was then carried out using the TruSeq Stranded mRNA assay (Illumina). In brief, this involved purifying polyadenylated mRNA from 0.1 to 4 mg of total RNA, using poly-T, oligonucleotide-attached magnetic beads. The mRNA was then fragmented by divalent cations, before being reverse transcribed into first-strand cDNA using random primers. DNA polymerase I and RNase H were then used to synthesize the second-strand cDNA. Adapters were then ligated to the cDNA fragments, and the products were purified and enriched by PCR to generate the final cDNA library. Samples were then paired-end sequenced using an Illumina HiSeq 2500 instrument (100 plus 100 cycles, plus indices). We sequenced an average of 22 million reads per library and aligned them to the human reference genome (hs37d5) using TopHat (release 2.1.0) (47) with default parameters. mRNA expression levels were calculated per gene using htseq-count (release 0.6.0) (48) with option “-s reverse -i gene_id -m intersection-nonempty” against release 19 of the comprehensive genome annotation from GENCODE. The counts were normalized to adjust for the difference in total number of counts associated with each sample and pooled across triplicates. The analysis focused on genes that were differentially expressed by a fold change of 2 or 5 in comparison with the unstimulated control. The average normalized RNA expression values determined by RNA-seq for annotated regions of the genome are presented in Supplemental Table I together with the fold induction values determined after stimulation.

### Public access to sequence data

All high-throughput sequencing data generated by this study are available at Gene Expression Omnibus (https://www.ncbi.nlm.nih.gov/geo/query/acc.cgi?acc=GSE90718) accession number GSE90718.

### RNA analysis by NanoString

RNA was extracted from Jurkat T cells as outlined above, either untreated or stimulated with PMA/ionomycin for 3 h. RNA was assayed using a custom-made human nCounter kit (NanoString Technologies), which contained a CodeSet of capture and reporter probes from 49 genes, identified by the RNA-seq analyses, including cytokines, chemokines, TFs, and surface markers associated with T cell activation (NanoString data included as Supplemental Table II). One hundred twenty-five nanograms of RNA was used in each CodeSet hybridization reaction. NanoString data were analyzed using nSolver analysis software v1.1 (NanoString Technologies). The results were normalized to the level of housekeeping genes and positive controls included in the CodeSet, whereas negative controls were subtracted from the normalized data. The data were then presented as the average of three biological replicates and plotted against data obtained via RNA-seq.

### RNA-seq correlations to public data

To directly compare our RNA-seq data with public data, raw fastq data from human T effector memory cells (TEMs) with and without 150-min CD3 plus CD28 stimulation (49) were retrieved from Gene Expression Omnibus series accession numbers GSM2370626 (resting) and GSM2370628 (with 150-min CD3 plus CD28), converted from SRA to fastq with the SRA Toolkit fastq-dump using the split-files option to obtain paired-end fastq files. Reads were subsequently aligned to the hs37d5 release of the human genome using TopHat, as in this study. To obtain comparable gene expression levels, normalized fragments per kilobase of transcript per million mapped reads counts, using bam files from TEMs with and without 150-min CD3 plus CD28 stimulation together with those from our study, were obtained using cuffnorm (50). For scatter plots, the genes.fpkm_table was read and log_2_ transformed in R, with counts plotted with the plot function. Pearson correlation coefficients were obtained using the cor function. TEM 2-fold–inducible genes were defined as those showing a fold change >1 × log_2_ with CD3 plus CD28 stimulation. For gene set enrichment analysis (GSEA), the GSEA package (51) was employed using a GSEA-readable version of the genes.fpkm_table as the expression dataset, the list of TEM 2-fold–inducible genes as the gene set group file, as well as a phenotype file describing the conditions used (Jurkat, Jurkat plus PMA/ionomycin). Parameters used were classic enrichment, average of probes, official gene symbol chip dataset, and gene-set permutation.

### ATAC-seq

To profile open chromatin, we modified the previously published ATAC-seq protocol (43): to isolate the Jurkat T cell nuclei, 50,000 cells were pelleted by centrifuging at 500 × *g* and 4°C for 10 min. Cells were then washed with 50 μl of cold PBS before being pelleted again as outlined above. Pellets were then resuspended in 500 μl of Nuclei EZ lysis buffer (Sigma-Aldrich) and placed on ice for 10 min. Finally, cells were pelleted by centrifugation at 500 × *g* and 4°C for 10 min before the supernatant was discarded. The isolated nuclei were then resuspended in a 50-μl reaction buffer containing 5 μl of Tn5 transposase and 25 μl of TD buffer (Nextera sample preparation kit from Illumina) and incubated at 37°C for 1 h. The DNA was then purified using DNA Clean & Concentrator (Zymo Research) and eluted into 23 μl of elution buffer. For library amplification, 20 μl of DNA was combined with 5 μl of indexing primers (Nextera sample preparation kit), 15 μl of Nextera PCR master mix, and 5 μl of Nextera PCR primer cocktail. DNA was then amplified for 15 cycles to enrich the tagmented DNA fragments. A PCR clean-up was then performed using AMPure XP beads (Beckman Coulter), and the small fragments were then resuspended in 32.5 μl of resuspension buffer (provided in the Nextera kit). DNA was quantified using a Qubit fluorometer (Life Technologies), and library sizes were then determined using TapeStation (Agilent Technologies). Sequencing was performed using a HiSeq 2500 to obtain an average of 60 million reads per sample.

### Alignment of ATAC-seq data

Trimmomatic (release 0.33) (52) was used in paired end mode to trim the adaptor sequence and separate sequences where both read ends were retained from sequences where only a single read was retained. Reads that aligned to the mitochondrial genome were removed in Bowtie 2 (release 2.2.5) (53). Bowtie 2 was then used first to align the trimmed paired-end data and then the single-ended read data to the hs37d5 reference genome. ATAC-seq peaks were identified using CisGenome (54) in one-sample mode based on reads from combined PMA and ionomycin treatment with options “-w 100 -s 25 -c 15 -g 0 -l 0 -br 0 -ssf 0.” Peak sizes were calculated by determining the number of fragment ends that were located within each peak for each of the samples separately. ATAC data are available at Gene Expression Omnibus (accession no. GSE90718) and are summarized in Supplemental Table III.

### Peak detection and filtering; coverage track generation

Replicate bam files were first merged using samtools merge (55). Peak detection and coverage track generation were subsequently performed via MACS (56) on merged replicates, using –keep-dup = auto -g hs -w -S. Peak and summit filtering was performed as previously described (57). Regions intersecting with regions previously published as blacklisted (58) as well as with simple repeats were excluded using bedtools intersect with -v as a parameter (59). For screenshots, coverage track files were uploaded to the University of California Santa Cruz Genome Browser (60).

### Two-way fold change analysis

Two-way combinations of filtered summit files were merged using the following bash command: cat < summits_A > .bed < summits_B > .bed | bedtools sort -i - | bedtools merge -i - -d 200. Tag counts for each experiment were retrieved for merged summits using the annotatePeaks function of the Homer package (61) using -hist 10 -ghist -wig as parameters. Tag counts were normalized via center scaling and subsequently sorted on log_2_ treatment/control fold change. Heat map images were obtained using Java TreeView (62). Annotation to the closest gene was performed using bedtools closest using the hg19 refFlat annotation, with -t first as a parameter.

### Motif discovery and heat maps

Motif discovery was performed with the findMotifsGenome function of Homer on regions showing enrichment ≥2 log_2_-fold change versus control, using default parameters. For isolation of the NFAT/AP-1 motif, motif discovery was performed on PMA plus ionomycin regions ≥2 log_2_-fold change versus control, using -len 16 as a parameter for findMotifsGenome. For motif heat maps, mapping of motifs was performed using the annotatePeaks function of Homer, using -hist 10 -ghist -size 2000 -m as parameters. Heat map images were obtained using Java TreeView. For motif discovery for the NFAT/AP-1 motif in 2× inducible human T-blast peaks, motif discovery was performed using Homer findMotifsGenome using -len 16 as a parameter to first look for 16-bp motifs. Motif optimization was then performed using Homer findMotifsGenome -len 20 -opt <16 bp motif >, as the original 16-bp motif isolated was offset.

### Digital genomic footprinting profiles

To assess motif occupancy by TFs, average ATAC insertion profiles around motifs identified from ATAC-seq peaks were retrieved using the dnase_average_profiles function of the Wellington package (63), using -A -n as parameters, -n performing normalization, and -A causing shifting of the reference genome coordinates by −4 and +5 bp, resulting in a shift of +4 and −5 bp as originally described for ATAC-seq footprinting (43).

### Venn diagrams

For ATAC-seq, Venn diagrams were obtained using pybedtools (64). For RNA-seq, BioVenn was used (65).

### ATAC correlations to public histone H3K27Ac chromatin immunoprecipitation sequencing data from primary T cells

Jurkat ATAC data were compared with published histone H3K27Ac chromatin immunoprecipitation sequencing data from stimulated human T cells. Raw H3K27Ac data from resting CD4^+^CD25^−^ and PMA/ionomycin-stimulated CD4^+^CD25^−^IL-17^−^ PBMC Th cells from the epigenome roadmap (66) were retrieved from Gene Expression Omnibus accession numbers GSM997239 and GSM772905, respectively. Reads were aligned to the hg19 genome assembly via Bowtie 2 to the bam format using the –very-sensitive-local parameter. Peak detection and coverage track generation was performed using macs14 using the –keep-dup = auto, g = hs, -w -S switches. A fold change analysis was carried out to determine 2× PMA/ionomycin enriched peaks versus resting. To map these peaks back onto the Jurkat PMA/ionomycin versus resting fold change, a left outer join was performed using coordinates sorted by increasing Jurkat PMA/ionomycin versus resting fold change and 2× PMA/ionomycin inducible H3K27ac peaks, using bedtools intersect with -loj as a parameter. Heat map images were obtained using Java TreeView.

### DNase I hypersensitive site analysis in human T cells

DNase sequencing (DNase-seq) was performed on previously described DNA samples purified from DNase I–digested chromatin derived from actively dividing human peripheral blood T cells (67). This material was generated from PBMCs that had been first stimulated with PHA for 2 d to generate T lymphoblasts, and then cultured for several cell cycles in the presence of recombinant human IL-2 as previously described (67). Cells were then either left untreated or stimulated for 5 h with 20 ng/ml PMA plus 2 μM calcium ionophore A23187 before performing DNase I digestions as previously described (67). DNase-seq libraries were prepared from ∼100- to 150-bp fragments as previously described (1).

### DNase-seq data processing

Using the Galaxy interface (68), single-ended DNAse-seq reads were aligned to the hg19 genome assembly via Bowtie 2 to the bam format using the –very-sensitive-local parameter. Peak detection and coverage track generation was performed using macs2 using the –keep-dup = auto, g = hs, -w -S switches. Fold change analysis was carried out to identify 2-fold PMA/ionomycin-enriched peaks relative to untreated controls. Raw DNase-seq data were deposited on Gene Expression Omnibus under accession GSE100418.

### Luciferase reporter gene assays of DHSs

Inducible DHSs were tested for inducible enhancer function using the previously described firefly luciferase reporter gene plasmid pTK229 (69). DNA fragments spanning each DHS were amplified by PCR from Jurkat T cell genomic DNA using primers with adapter sequences designed for cloning into the BamHI and XhoI sites upstream of the herpes simplex thymidine kinase promoter in pTK229. The primers had the following sequences, with the adapter portion shown in lowercase: *GEM* upstream DHS, 5′-taagggatccCGCTTGTCAGGGCATCATTTTCT-3′ and 5′-cttactcgagAATGGAACTGGTGGCCTGCC-3′; *GEM* intron DHS, 5′-taagggatccACACTGGCCCTATTTCTCCCT-3′ and 5′-cttactcgagCCACAGCTTCAGTCTGAGCGT-3′; *DUSP5*, 5′-taagggatccATTGAAGGCTGCCGTGAGTG-3′ and 5′-cttactcgagTTCTGGGTGGGGTGTTGGGT-3′; *ZBTB16*, 5′-taagggatccTGCCTCCTCGACAGCTCAGA-3′ and 5′-cttactcgagACTTCCCACCATACCCAGTTCT-3′. As an additional control we employed a previously described plasmid that includes the *IL3* −37 kb enhancer cloned as an XbaI fragment (X37) into pTK229 (16). Plasmids were assayed in Jurkat T cells by transient transfection assay using the dual luciferase method, together with the *Renilla* firefly control plasmid pRL-TK, as previously described (16). Transfected cells were cultured for 24 h and then stimulated for 8 h with 20 ng/ml PMA and/or 2 μM calcium ionophore A23187, before harvesting cell lysates for assay using a Dual-Luciferase kit (Promega).

## Results

### Uncoupling of the kinase and Ca^2+^ signaling networks using stimulation via just PMA or ionomycin alone

To investigate the gene regulation networks downstream of TCR signaling (Fig. 1A) we used either PMA or the Ca^2+^ ionophore ionomycin alone to stimulate just the PKC- or calcineurin-mediated pathways in Jurkat T cells. In agreement with previous analyses (70–72), stimulation with PMA alone resulted in NF-κB pathway activation (as visualized by stimuli-induced p65 phosphorylation) whereas stimulation with ionomycin alone led to NFAT activation (as exhibited by NFATc2 dephosphorylation, resulting in a faster migrating protein) (Fig. 1C, 1D). PMA stimulation in the absence of calcineurin activity is also known to be a strong inducer of AP-1 in Jurkat cells (18).

To investigate transcriptional responses to PMA and ionomycin, we employed Jurkat T cells transfected with luciferase reporter gene lentiviruses, driven by either the full-length NFAT/AP-1–dependent IL-2 promoter (IL-2–Luc) or an array of five NF-κB sites (5κB-Luc). Combined PMA and ionomycin treatment resulted in activation of both NF-κB and NFAT/AP-1–dependent transcription, peaking after ∼8–10 h (Fig. 1E, 1F). In Jurkat cells this treatment also led to de novo production of secreted IL-2 protein (Fig. 1G), a major growth factor involved in clonal expansion and a well-established marker of T cell activation (13, 14). PMA or ionomycin treatment alone did not elicit an IL-2 promoter response, confirming that Ca^2+^ and PKC costimulation is required for robust IL-2 promoter activation in T cells. These results mirror those seen previously for the NFAT/AP-1–dependent IL-3 and GM-CSF (*CSF2*) genes in Jurkat T cells (18). PMA alone did, however, induce a modest NF-κB–dependent response (Fig. 1E).

### Distinct gene expression patterns are associated with ionomycin and/or PMA stimulation

**FIGURE 2.**
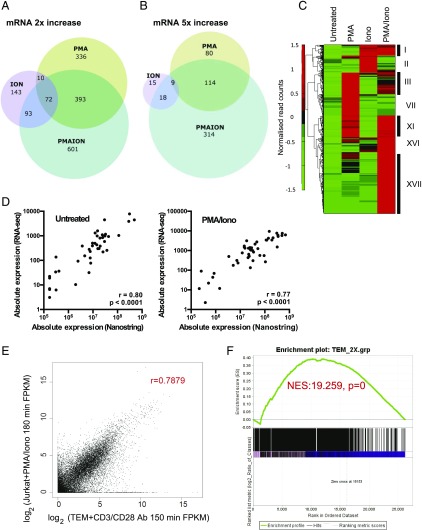
Distinct gene expression patterns are associated with PMA and/or ionomycin stimulation. (**A** and **B**) Venn diagrams showing the overlaps between PMA-, ionomycin-, and PMA/ionomycin-regulated genes in Jurkat cells for genes that are upregulated by at least 2-fold (A) or 5-fold (B) by each condition relative to untreated cells. (**C**) Hierarchical clustering analysis of PMA, ionomycin, or PMA/ionomycin-regulated genes from (A). Data are presented as a heat map showing normalized read counts across rows. Different clusters are depicted with roman numerals. (**D**) Comparison of mRNA levels determined by RNA-seq of untreated and PMA/ionomycin-stimulated Jurkat T cells, using the above data, compared with RNA analyses of a subset of genes by Nanostring (using the same treatment protocol). (**E**) Comparison of mRNA levels determined by RNA-seq of stimulated Jurkat T cells, using the above data, compared with stimulated TEM cells using published data (49). The Jurkat T cells were stimulated for 3 h with PMA and ionomycin as above, whereas TEMs were stimulated for 150 min with CD3 and CD28 Abs (49). (**F**) GSEA was performed using genes that are 2-fold induced in TEMs by CD3 and CD28 Abs, compared with genes sorted according to the level of induction in PMA/ionomycin-stimulated Jurkat cells. All genome-wide RNA-seq data are available in Supplemental Table I, which lists the averages of three independent RNA-seq values, and the fold change in response to specific stimuli. Nanostring data for mRNA expression are available in Supplemental Table II.

To define specific gene targets downstream of pathways induced by PMA and/or ionomycin, and to investigate correlations between chomatin structure changes and inducible gene expression, we characterized global mRNA profiles and chromatin accesibility patterns using RNA and ATAC sequencing. We identified 1648 genes whose expression was increased by ≥2-fold by at least one treatment in comparison with untreated cells (Fig. 2A). There were 1159 genes activated 2-fold by the combined treatment, and, of these, 455 were activated by ≥5-fold (Fig. 2A, 2B, Supplemental Table I). Overall, genes upregulated upon ionomycin or PMA treatment alone showed little overlap. The combined treatment resulted in upregulation of many genes that were activated by a single stimulus, plus numerous genes not seen with either treatment alone.

**FIGURE 3.**
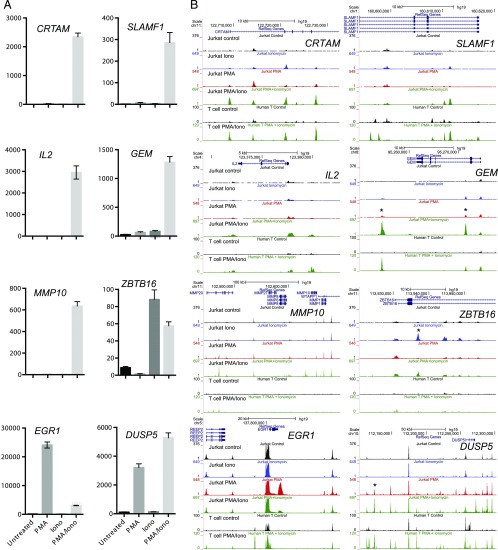
Representative gene expression patterns and corresponding chromatin changes. (**A**) Bar graphs of individual gene expression patterns determined by RNA-seq and measured in fragments per kilobase of transcript per million mapped reads. Error bars indicate SDs based on triplicates. Shown are representative patterns observed in the data. (**B**) University of California Santa Cruz Genome Browser shots of the ATAC-seq patterns present in Jurkat T cells for different gene loci following treatment with 20 ng/ml PMA, 1 μg/ml ionomycin, or PMA and ionomycin (20 ng/ml PMA/1 μg/ml ionomycin) for 3 h, and DNase-seq profiles of human T lymphoblastoid cells before and after treatment with 20 ng/ml PMA plus 2 μM calcium ionophore A23187 for 5 h.

Hierarchical clustering confirmed that most of the 1648 upregulated genes were activated by the combined PMA and ionomycin stimulation (groups I, XI, XVI, and XVII), and out of 1159 genes activated 2-fold, 52% were uniquely regulated by the combined treatment (Fig. 2C) (see group XVII for uniquely regulated genes). This gene set includes key inflammatory cytokines and growth factors such as *TNF*, *IL2*, *IL3*, and *CSF2* (13, 14, 37) and other immune mediators such as *CRTAM*, *SLAMF1*, *MMP10*, and *GEM* (Fig. 3A, Supplemental Table I). The expression patterns of these genes are consistent with the requirement for synergistic activation via kinase and Ca^2+^ signaling.

A large number of genes were also activated by PMA treatment alone (group VII), with 811 genes upregulated by 2-fold and 203 of these by 5-fold (Fig. 2A, 2B, Supplemental Table I). Most of these were a subset of the PMA/ionomycin-inducible genes (total of 393, group XI) (Fig. 2C). These included *DUSP5*, which was induced by PMA alone but was activated to a greater extent by costimulation with ionomycin (Fig. 3A). However, it also included inducible genes such as the TF gene *EGR1*, a known regulator of the IL-2 promoter (73), which was activated by PMA but partially suppressed by costimulation with ionomycin (Fig. 3A). As PMA alone induced a similar subset of TFs to proinflammatory cytokines, including NF-κB and AP-1, the PMA-inducible genes were compared with genes that were induced by TNF (Table I). Out of a total of 36 genes that were induced at least 5-fold by TNF, we identified 27 that were also induced at least 2-fold by PMA, with 24 of these being induced by at least 5-fold. Many of these genes function as immune regulators, including two members of the NF-κB family of TFs (*NFKB2* and *RELB*), as well as the NF-κB inhibitor *NFKBIA* (Table I).

**Table I.** Genes regulated by the PMA and TNF treatment

	TNF Fold Change	PMA Fold Change
MIR146A	40.5	46.1
WNT10A	30.9	11.5
CHRNA6	25.1	3.0
CCL1	17.3	11.9
BIRC3	16.2	14.9
BCL3	16.1	15.7
RELB	14.7	16.5
IL-4I1	13.9	11.7
NFKBIA	13.1	5.8
LTB	11.9	46.1
KREMEN2	8.2	4.2
NFKB2	8.0	6.8
PGLYRP4	7.7	17.2
RGS16	7.3	26.8
GATA3-AS1	7.3	16.4
EPHA2	6.8	6.7
IER3	6.5	92.8
TTC40	6.2	4.2
TNFSF10	5.8	6.4
NINJ1	5.7	7.7
TNFAIP3	5.7	7.3
SGK1	5.6	23.6
FCGBP	5.6	3.9
CYTH4	5.6	8.2
PRR9	5.4	21.7
TNFRSF18	5.3	25.6
TRAF4	5.1	5.9
PGLYRP3	5.1	6.5

Subset of 28 of the 36 genes induced at least 5-fold by TNF, which are also induced at least 2-fold by PMA, showing the fold induction in Jurkat T cells compared with nonstimulated cells. This list includes 24 genes induced at least 5-fold by PMA. Jurkat cells were stimulated with 30 ng/ml TNF for 3 h.

A group of 318 genes was induced by at least 2-fold by the Ca^2+^ signaling pathway via ionomycin alone (Fig. 2A, Supplemental Table I). Of these, just 42 genes were upregulated by ≥5-fold, which was far fewer than the number of genes induced by PMA alone (Fig. 2B). These included the TF PLZF encoded by *ZBTB16* (Fig. 3A). This observation is consistent with previous observations that NFAT activation on its own is more strongly associated with chromatin remodeling than with transcriptional activation (15). These results suggest that in vitro activation of the full complement of TCR-responsive genes is dependent on both PMA and ionomycin and amounts to more than just the sum of both.

To confirm the validity of the RNA-seq data, we compared a subset of the above data with values obtained by Nanostring analyses. For both untreated and PMA/ionomycin-stimulated cells, the Nanostring values correlated well with the RNA-seq values (Fig. 2D, Supplemental Table II).

To confirm that Jurkat T cells represented a valid model for studying the PMA/ionomycin induciblity of genes in T lineage cells, we compared the above data obtained from stimulated Jurkat cells with published data obtained from primary human TEMs stimulated using CD3 and CD28 Abs (Fig. 2E) (49). This analysis demonstrated that there was a good correlation between the two data sets. These conclusions were further supported by GSEA comparing the two data sets (Fig. 2F).

### Stimulus-specific gene expression patterns correlate with chromatin landscapes

To link changes in chromatin structure with inducible gene expression profiles, we analyzed ATAC-seq profiles that were collected simultaneously with the RNA-seq analyses. ATAC-seq is a surrogate for DHS analysis and it is similarly used to identify accessible regions of DNA. Accessible DNA regions are free from nucleosomes within chromatin, and they are typically occupied by TFs in place of histones (43). Typical ATAC-seq profiles are shown in Fig. 3B, where it can be seen that additional ATAC peaks are acquired in inducible loci in patterns that mirror the patterns of mRNA induction seen in Fig. 3A. For *CRTAM*, *SLAMF1*, *MMP10*, and *GEM* there are prominent peaks that are induced via the combined PMA and ionomycin treatment, but not in response to either agent alone. This pattern is consistent with cooperation between NFAT and AP-1. For the ionomycin-inducible *ZBTB16* gene there is an ionomycin-induced ATAC peak. For the PMA-inducible *DUSP5* gene there are two PMA-inducible ATAC peaks, one of which is further induced upon addition of ionomycin, in parallel with *DUSP5* mRNA. In contrast, the PMA-inducible *EGR1* gene also exhibits a PMA-induced ATAC peak, but in this case the peak is suppressed by the addition of ionomycin, in parallel with suppression of mRNA expression.

To determine whether the above ATAC data were representative of rapidly proliferating primary human T cells, we performed a DNase I sequencing analysis of DHSs present in bulk human T lymphoblastoid cells, generated by first stimulating human peripheral blood T cells with PHA and then expanding them by culture in the presence of IL-2 (67). This confirmed that most of the ATAC peaks induced by PMA/ionomycin in Jurkat T cells were also induced by the same stimuli in nontransformed proliferating human T cells (Fig. 3B). Interestingly, a cluster of metallopeptidase genes encompassed an array of ATAC peaks spanning *MMP10* that were induced in Jurkat T cells but not in primary human T cells.

**FIGURE 4.**
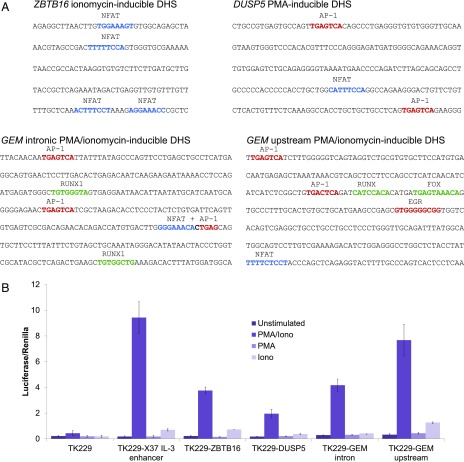
Analyses of sequences found at inducible ATAC peaks. (**A**) Inducible NFAT, AP-1, and or EGR binding motifs are present in the sequences corresponding to stimulus-specific ATAC-seq peaks. Shown are the analyses of inducible ATAC peaks from the PMA/ionomycin-inducible GEM locus, the ionomycin-inducible ZBTB16 locus, and the PMA-inducible DUSP5 locus (corresponding peaks marked by asterisks in Fig. 3B). (**B**) Luciferase reporter gene analysis of inducible enhancer function for the above sequences from ZBT16, DUSP5, and GEM genes, plus the inducible human −37 kb IL-3 gene enhancer (16) used in this study as a positive control.

An analysis of the sequences of four of the inducible ATAC peaks (marked by asterisks in Fig. 3B) showed that two PMA/ionomycin-inducible peaks in the *GEM* locus contained both NFAT and AP-1 motifs, an ionomycin-induced ATAC peak in *ZBTB16* contained four NFAT motifs, whereas a PMA-induced peak near *EGR1* contained two AP-1 motifs and one NFAT motif, which can account for the observed responses of these elements (Fig. 4A). These four regions were also tested for enhancer activity in transient transfection assays of luciferase reporter genes in Jurkat T cells, using the PMA/ionomycin-inducible human −37 kb IL-3 enhancer (16) as a control (Fig. 4B). These analyses demonstrated that each region functions as an inducible enhancer dependent on both PMA and ionomycin. These findings revealed that enhancer activity still required both calcium and kinase signaling, even in instances when the corresponding ATAC peak was induced by either PMA or ionomycin alone.

### Global analysis of ATAC and mRNA profiles

**FIGURE 5.**
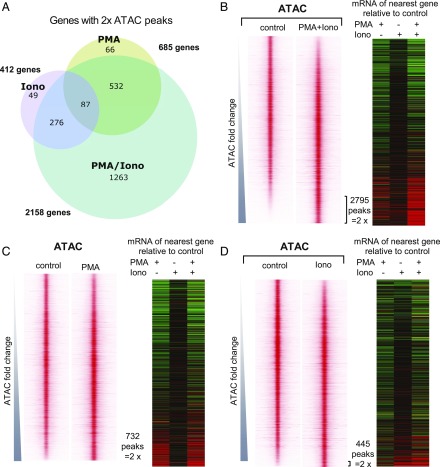
PMA and/or ionomycin treatments induce distinct chromatin changes. (**A**) Venn diagram showing differential chromatin regulation in Jurkat T cells treated with PMA (20 ng/ml), ionomycin (1 μg/ml), or PMA and ionomycin (20 ng/ml PMA/1 μg/ml ionomycin) cotreatment. Shown are the numbers of genes associated with ATAC-seq peaks that are upregulated by >2-fold by each condition relative to untreated cells. (**B**–**D**) Profiles of the ATAC-seq signals within 2-kb windows centered on each peak for PMA/ionomycin (B), PMA (C), and ionomycin (D), aligned with the mRNA expression levels (fold change relative to the untreated control) of the nearest gene (defined by the proximity between ATAC-seq peak and the nearest transcription start site). Window-centered analysis includes the union of all peaks present in each treatment. Peaks are displayed in the order of increasing ATAC-seq signal for each treatment relative to the untreated control. All Jurkat T cell ATAC peak data are available in Supplemental Table III, which lists the averages of three independent ATAC values, and the fold change in response to specific stimuli.

As was seen above at the mRNA level, stimulation with PMA or ionomycin alone induced far fewer specific ATAC peaks than were induced by PMA plus ionomycin (Fig. 5). We identified 2795 ATAC peaks that were induced in Jurkat cells by PMA and ionomycin at least 2-fold, which were associated with 2158 genes. The profiles of all of the 19,763 peaks identified in untreated or PMA/ionomycin-stimulated Jurkat cells (Supplemental Table III) are depicted in Fig. 5B, which shows the ATAC peak profiles centered within 2-kb regions and ranked in order of increasing inducibility. PMA induced 732 peaks 2-fold, and these were associated with 685 genes, whereas ionomycin induced 445 peaks 2-fold, which were associated with 412 genes. The vast majority of the genes with peaks induced by PMA or ionomycin alone also have peaks induced by the combined treatment (Fig. 5A). Our results suggest that in vitro activation of TCR-responsive enhancers requires treatment with both PMA and ionomycin, resulting in the activation of regulatory elements not induced by either alone. The PMA/ionomycin-inducible peaks represented 14% of the total number of ATAC peaks detected in either stimulated or nonstimulated Jurkat cells.

To correlate the ATAC data with the mRNA data, we depicted the inducible responses of the genes nearest to the ATAC peaks as heat maps, showing the relative fold changes in expression in response to the three different treatments (Fig. 5B–D). These analyses reinforced the view that the combination of PMA and ionomycin was a much stonger inducing agent than ionomycin alone, whereas PMA alone was sufficient to activate a larger subset of genes than ionomycin alone.

**FIGURE 6.**
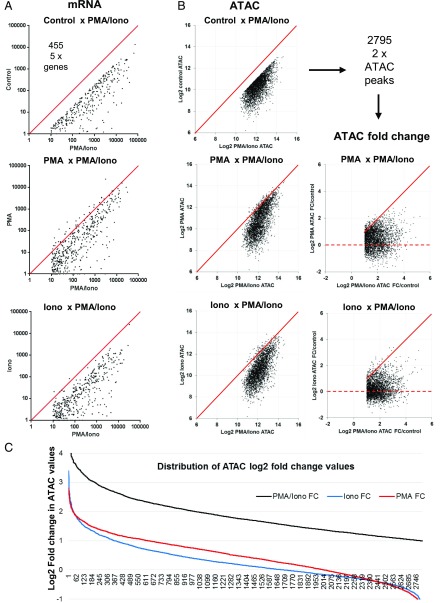
PMA/ionomycin-induced gene expression patterns are correlated with chromatin changes. (**A**) Comparison of individual inducible reponses to the single and combined stimuli for each of the 455 genes upregulated (by 5-fold) by PMA/ionomycin cotreatment (as defined in Fig. 2B). Shown are scatter plots of individual gene expression values across different treatment conditions. A line representing identical values is shown in red. (**B**) Differential analysis of the 2795 ATAC-seq peaks that were induced by PMA and ionomycin cotreatment by 2-fold (as defined in Fig. 4A). Shown are scatter plots of individual ATAC-seq peak values across different treatment conditions. Data are represented either as peak intensity or fold change (FC) in log_2_ scale. A line representing identical values is shown in red. The broken line indicates the level of no fold change. (**C**) Distribution of ATAC-seq peak values across different treatment conditions (in log_2_).

To track the responses of individual genes, we directly compared the individual inducible responses to the single and combined stimuli for each of the 455 genes where mRNA was induced 5-fold (Fig. 6A), and for each of the 2795 ATAC peaks that were induced 2-fold (Fig. 6B) by the combined stimulation with PMA and ionomycin. The mRNA responses to PMA revealed a trend for the whole population of 455 inducible genes to show partial responses to PMA alone, but most of these had much stronger responses to the combined treatment. In contrast, the pattern of mRNA responses to ionomycin more closely resembled that seen for unstimulated cells. The ATAC data showed a similar trend whereby PMA, but not ionomycin, induced partial responses at many chromatin regions. The fold-change analyses in Fig. 6B and 6C also showed that many of the ATAC peaks were partially induced by PMA alone, and to a greater extent than by ionomycin alone. The magnitudes of the changes induced in ATAC peaks by ionomycin were mostly within the margins of error, confirming that ionomycin alone is largely ineffective at inducing either chromatin accessibility or mRNA expression.

### Enriched TF binding motifs reflect T cell activation markers

**FIGURE 7.**
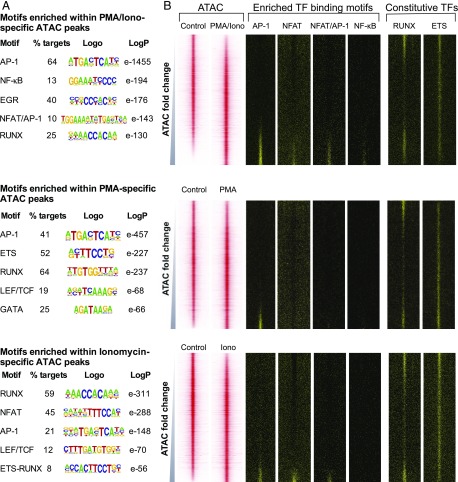
Differential regulation of the chromatin landscape. (**A**) Result of de novo TF binding motif search of PMA-specific (top), ionomycin-specific (middle), or PMA/ionomycin-specific (bottom) ATAC-seq peaks using HOMER. Top motifs characterized by the fraction of peaks present (per treatment), sequence logo, and *p* value for enrichment (versus randomized sequence control) are shown. (**B**) Profiles of the ATAC-seq signals within each 2-kb window centered on each peak for PMA, ionomycin, and PMA/ionomycin aligned with the locations of the specific TF binding motifs. Window-centered analysis includes the union of all peaks present in each treatment, with peaks displayed in order of increasing ATAC-seq signal for each treatment relative to the control.

To gain insights as to how simultaneous stimulation with PMA and ionomycin could result in activation of specific elements not induced by either treatment alone, we hypothesized that this might be due to these elements requiring binding of the full complement of TCR-induced TFs at the same time (i.e., AP-1, NFAT, and NF-κB), including the composite motif for AP-1 and NFAT (Fig. 1B). To identify TFs involved in treatment-specific chromatin landscapes, we used HOMER to perform an unbiased search for enriched TF binding motifs within accessible regions of chromatin (66) (Fig. 7A). Motifs identified in this way were then mapped over the coordinates of the DHS profiles depicted above in Figs. 5 and 7B. These analyses revealed the motif signatures of the NFAT, AP-1, EGR, and NF-κB families of TCR-inducible TFs in the PMA/ionomycin-inducible ATAC peaks (Fig. 7). The NFAT motifs were identified in this study as part of the previously described composite NFAT/AP-1 motif, which is known to integrate Ca^2+^ and kinase signaling pathways in T cells (11, 21, 24–26). The NFAT, AP-1, and NF-κB motifs were all preferentially enriched in the inducible ATAC peaks (Fig. 7B).

HOMER analysis of the PMA-inducible ATAC peaks revealed motif signatures for the kinase-inducible TF AP-1 (Fig. 7A); importantly, however, no NFAT motifs were seen in the DHS profiles of the PMA-inducible ATAC peaks (Fig. 7B). This reflects the conclusions above that PMA alone does indeed induce a subset of genes in the absence of Ca^2+^ signaling.

Curiously, HOMER analysis of the ionomycin-inducible ATAC peaks revealed motif signatures for both AP-1 and NFAT. Hence, AP-1 motifs are enriched in DHSs induced by Ca^2+^ signaling even in the absence of AP-1. We interpreted this as meaning that NFAT motifs are rarely found in inducible DHSs in T cells in this absence of its usual partner AP-1, and that NFAT rarely functions to activate gene expression on its own. However, in some cases it is clear that NFAT is sufficient to induce DHSs in the absence of AP-1, presumably at sites that encompass a sufficient number of high-affinity motifs, for example the DHS defined above in the *ZBTB16* locus (Fig. 4A).

The inducible DHSs were also enriched to varying degrees in RUNX and ETS motifs, which are sometimes observed as composite ETS/RUNX motifs (1, 60) and are known to function widely to regulate gene expression in T cells and at other stages of blood cell development (1, 74, 75). Inducible DHSs also contain motifs for TCF/LEF family TFs (76). However, these constitutive TFs were more enriched in PMA- or ionomycin-specific peaks than in PMA/ionomycin-specific sites, suggesting that the latter are truly inducible sites. Overall, our results suggest that the activation of TCR-inducible enhancers requires binding of both AP-1 and NFAT, either to distinct motifs or as a composite motif, as well as NF-κB binding.

**FIGURE 8.**
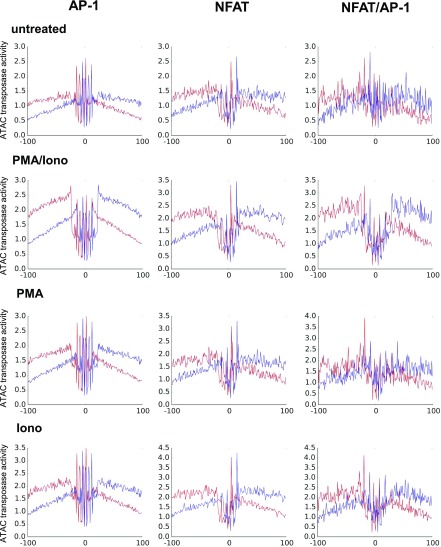
Digital genomic footprinting analyses (63) showing occupancy at genomic coordinates of AP-1, NFAT, and composite NFAT/AP-1 motifs identified in 2-fold–induced ATAC peaks in untreated Jurkat cells and Jurkat cells stimulated with PMA plus ionomycin, PMA alone, or ionomycin alone. Upper strand reads are shown in red and lower strand reads are shown in blue.

To find evidence directly linking inducible chromatin accessibility with TF activity, we used the Wellington algorithm (63) to perform a digital footprinting analysis of occupied TF binding motifs. We centered the average ATAC transposase insertion profiles at genomic coordinates of specific motifs identified in PMA/ionomycin-inducible peaks (Figs. 7, 8). This produced strong evidence that AP-1, NFAT, and NFAT/AP-1 motifs were indeed occupied within the ATAC peaks in cells stimulated with PMA and ionomycin. These data also suggested that stable AP-1 and NFAT binding, as well as chromatin opening, are highly cooperative processes relying on both kinase and calcium signaling, because much less evidence of occupancy was detected after stimulation with either PMA or ionophore alone (Fig. 8).

### Jurkat T cells and primary human T cells share a common set of inducible DNA elements

**FIGURE 9.**
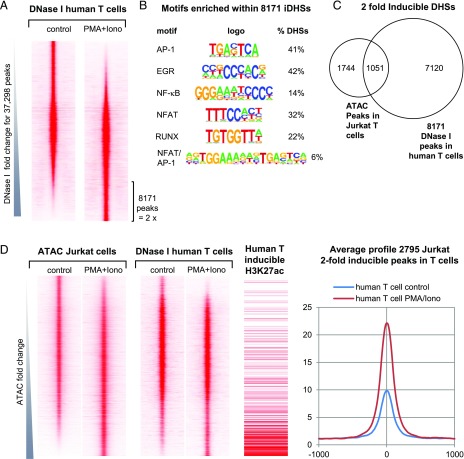
DHS analyses of human T cells stimulated with PMA and calcium ionophore. (**A**) DNase-seq profiles of actively dividing human T blast cells before and after stimulation with PMA and A23187, ranked in order of increasing inducibility. (**B**) Results of de novo TF binding motif search for the 8171 DHS peaks induced 2-fold in human T blast cells determined using HOMER. (**C**) Venn diagram showing the overlap between 8171 DHS peaks induced 2-fold in human T blast cells compared with 2795 ATAC peaks induced 2-fold in Jurkat T cells. (**D**) Side-by-side comparison of profiles of DHS peaks present in stimulated human T blast cells on the same axis alongside the Jurkat T cell ATAC peaks ranked as in Fig. 7B for Jurkat cells stimulated with PMA and ionomycin. Also shown alongside are 1) the locations of inducible histone H3K27Ac regions as identified from published data from resting and stimulated human CD4 T cells (66), and 2) the average profiles for the 2795 peaks defined as inducible in Jurkat cells in (D) using values from human T blast cells before and after stimulation with PMA and A23187 as in (A).

A global analysis of the PMA/ionomycin-inducible DHSs identified in human T blast cells revealed 8171 DHSs that were induced 2-fold relative to untreated cells (Fig. 9A). These inducible DHSs had a similar TF motif composition to the above PMA/ionomycin-inducible Jurkat ATAC peaks (Fig. 7A), and to the PMA/ionomycin-inducible DHSs present in mouse T blast cells (1) (Fig. 9C). We found that 1051 of these DNA elements were inducible in both Jurkat T cells and in primary T cells (Fig. 9C).

We next aligned the two sets of human T blast cell DHS data with the ATAC data from Figs. 5B and 9D. This confirmed that many of the ATAC peaks induced in Jurkat cells were also strongly induced in primary T cells. This conclusion was further supported by 1) aligning the Jurkat ATAC data with published histone H3K27Ac chromatin immunoprecipitation sequencing data from stimulated human CD4 T cells, showing the relative fold increase in signal in response to stimulation, and 2) the average DHS profiles from the above human T cell data for of the of 2795 inducible ATAC peaks found in Jurkat T cells (Fig. 9D).

## Discussion

This study has confirmed the long-held view that integration of Ca^2+^ and kinase signaling is needed for efficient activation of inducible gene expression in T cells. We found that for many immune response genes, both signals are needed to open up the chromatin and activate transcription. However, the immune system encompasses other genes that are induced in a range of cell types, by a wide variety of other receptors linked to kinase but not Ca^2+^ signaling pathways. These include proinflammatory receptors, such as Toll family receptors, and receptors that respond to cytokines such as IL-1 and TNF, which also lead to induction of the TFs AP-1 and NF-κB. For these reasons it makes sense that we find a subset of genes that are induced by PMA via PKC-dependent pathways, in the absence of Ca^2+^ signaling. Significantly, we found that two-thirds of all of the genes induced 5-fold by TNF were also induced 5-fold by PMA (Table I). In contrast, there appear to be fewer gene-inducing signaling pathways linked to Ca^2+^ signaling in the absence of kinase signaling.

Previous studies have also found that cooperation between NFAT and AP-1 is required for efficient responses by most TCR-inducible genes. T cells lose the ability to efficiently activate TCR-inducible genes in cells expressing an engineered form of NFATc2, in which the AP-1–binding domain is mutated (41, 42). This cooperation is also dependent on the spatial relationship between the NFAT and AP-1 complexes (24). An example of this type of composite element is seen in the sequence 5′-GGGAAACACTGAGCAG-3′ in the *GEM* intronic enhancer in Fig. 4A, which has incomplete versions of the underlined NFAT and AP-1 motifs. As with similar elements in the *CSF2* enhancer (25), this enhancer probably relies on integration between calcium and kinase signaling because it is predicted to bind NFAT and AP-1 in a highly cooperative manner. It is also known that composite NFAT/AP-1 motifs that do not have the binding sites orientated in the precise arrangement depicted in Fig. 1B, such as the GM170 element in the *CSF2* enhancer, are found to have much less transactivation potential and fail to support cooperative binding of NFAT and AP-1 (25). Furthermore, it has also been proposed that NFAT and AP-1 can cooperate by supporting separate functions. A study of the GM420 high-affinity NFAT-binding site from the *CSF2* enhancer revealed that an array of three copies of just the NFAT site, without the adjacent AP-1 motif, was sufficient to create an inducible DHS but not sufficient to activate transcription of a linked reporter gene (15). This specific NFAT site was able to function as a strong activator of transcription only when it was linked to the AP-1 motif. However, the *CSF2* enhancer includes just one high-affinity NFAT, with two of the NFAT sites relying on AP-1 for efficient binding (25). In its natural context, the *CSF2* enhancer also requires cooperation between NFAT and AP-1 for efficient induction of a DHS, whereby calcium ionophore alone is a poor inducer (18). An analysis of TF occupancy also suggested that NFAT and AP-1 are very inefficient at binding independently when induced by either PMA or ionomycin alone (Fig. 8).

Our results also suggest that it is the synergistic action of inducible TFs acting together, including NFAT, AP-1, NF-κB, and EGR family proteins, that is responsible for opening up the chromatin at TCR-inducible enhancers, as well as the tightly regulated inducible properties of these elements. In rare cases, such as in the *ZBTB16* locus, several copies of just an NFAT motif are found, which are also sufficient to direct chromatin opening, as described above for an artificial array of strong NFAT sites. As observed in our previous study of activated mouse T cells (1), the inducible DHSs defined in this study were also less enriched in motifs for constitutive TFs, such as ETS and RUNX family proteins. In contrast, these motifs are known to be enriched in DNA elements that function as locus priming elements in previously activated T cells, and which exist as constitutive DHSs in Jurkat T cells (1, 69, 77). Similarly, RUNX and ETS motifs were found at a higher density in the PMA-inducible and ionomycin-inducible DHSs. This suggests that for many TCR-inducible DHSs, the required critical mass of TFs is not attained using either PMA or ionomycin alone, but can be attained when RUNX and ETS motifs are present at a sufficient density. Although some DHSs were observed to be strongly induced by just PMA or ionomycin alone (e.g., *ZBTB16*, *DUSP5*, and *EGR1*, Fig. 3B), most of the PMA-inducible mRNA changes were not associated with an inducible DHS change (e.g., *BIRC3*, *BCL3*, *RELB*, and *TNAIP3*) However, an examination of publicly available data (http://genome.cse.ucsc.edu/) revealed that these genes do contain numerous constitutive DHSs, which are present in Jurkat T cells but not in naive T cells (data not shown). This implies that much of the PMA response in Jurkat cells is mediated via constitutively open chromatin regions that recruit PMA-inducible factors. These regions most likely correspond to the priming elements previously defined in mouse T cells, which are maintained by ETS and RUNX factors (1).

Overall, this study demonstrates that tight regulation of most immune response genes is made possible by the obligate cooperation between Ca^2+^- and kinase-inducible factors acting at the level of chromatin, whereas a small subset of genes exist in a poised state able to respond to just a kinase signal.

## Supplementary Material

Data Supplement
